# A transgenic approach to study argininosuccinate synthetase gene expression

**DOI:** 10.1186/1423-0127-21-42

**Published:** 2014-05-13

**Authors:** Shih-Chang Shiue, Miao-Zeng Huang, Tsung-Sheng Su

**Affiliations:** 1 Institute of Microbiology & Immunology, National Yang-Ming University, 112 Taipei, Taiwan; 2 Department of Medical Research, Taipei Veterans General Hospital, 112 Taipei, Taiwan

**Keywords:** Argininosuccinate synthetase, Transgenic mouse, Bacterial artificial chromosome, Green fluorescence protein, Developmental regulation, Tissue-specific regulation, Post-transcriptional regulation

## Abstract

**Background:**

Argininosuccinate synthetase (ASS) participates in urea, nitric oxide and arginine production. Besides transcriptional regulation, a post-transcriptional regulation affecting nuclear precursor RNA stability has been reported. To study whether such post-transcriptional regulation underlines particular temporal and spatial ASS expression, and to investigate how human *ASS* gene behaves in a mouse background, a transgenic mouse system using a modified bacterial artificial chromosome carrying the human *ASS* gene tagged with EGFP was employed.

**Results:**

Two lines of *ASS-EGFP* transgenic mice were generated: one with *EGFP* under transcriptional control similar to that of the endogenous *ASS* gene, another with *EGFP* under both transcriptional and post-transcriptional regulation as that of the endogenous *ASS* mRNA. EGFP expression in the liver, the organ for urea production, and in the intestine and kidney that are responsible for arginine biosynthesis, was examined. Organs taken from embryos E14.5 stage to young adult were examined under a fluorescence microscope either directly or after cryosectioning. The levels of *EGFP* and endogenous mouse *Ass* mRNAs were also quantified by S1 nuclease mapping. EGFP fluorescence and *EGFP* mRNA levels in both the liver and kidney were found to increase progressively from embryonic stage toward birth. In contrast, EGFP expression in the intestine was higher in neonates and started to decline at about 3 weeks after birth. Comparison between the EGFP profiles of the two transgenic lines indicated the developmental and tissue-specific regulation was mainly controlled at the transcriptional level. The *ASS* transgene was of human origin. EGFP expression in the liver followed essentially the mouse *Ass* pattern as evidenced by zonation distribution of fluorescence and the level of *EGFP* mRNA at birth. However, in the small intestine, *Ass* mRNA level declined sharply at 3 week of age, and yet substantial *EGFP* mRNA was still detectable at this stage. Thus, the time course of *EGFP* expression in the transgenic mice resembled that of the human *ASS* gene.

**Conclusions:**

We demonstrate that the transgenic mouse system reported here has the merit of sensitivity and direct visualization advantage, and is ideal for annotating temporal and spatial expression profiles and the regulation mode of the *ASS* gene.

## Background

Argininosuccinate synthetase (ASS; EC 6.3.4.5) is an enzyme that functions in the catalysis of the conversion of citrulline and aspartate to argininosuccinate, which is further converted to arginine by argininosuccinate lyase [1,2]. ASS catalyzes the rate-limiting step in arginine biosynthesis. Arginine plays a role in the synthesis of urea, nitric oxide (NO), polyamines, proline, glutamate, creatine and agmatine [3]. Thus, ASS participates in fine-tuning production of NO and others to maintain cellular homeostasis in response to cellular and environmental stimuli. Conceivably, ASS, one of the key enzymes involving in arginine metabolism, is subjected to various mechanisms of regulation in both physiological and disease states.

Hormones, such as glucocorticoid, glucagon and insulin, are major regulators of the expression of urea cycle enzymes in the liver [1,2]. We have previously identified that the cAMP response element (CRE) located at about 10 kb upstream of the transcription start site of the human *ASS* gene is most likely the target site of the CRE-binding protein (CREB) to mediate glucagon action [4]. However, the mechanism by which glucocorticoid and insulin act on *ASS* expression remains unknown. On the other hand, *ASS* expression in non-hepatic cells were shown to be induced by interleukin-1β through NF-κB activation acting at a putative NF-κB binding site at the *ASS* promoter [5]. Moreover, the proximal promoter of the *ASS* gene was shown to contain an E-box recognized by c-Myc and HIF-1α, and a GC-box targeted by Sp4 where *ASS* expression involves interactions between the positive transcriptional factors c-Myc and Sp4 and the negative factor HIF-1α [6]. In addition to regulation at transcription initiation, we have also identified a novel post-transcriptional event affecting *ASS* nuclear precursor RNA stability in the canavanine-resistant variants of a human squamous cell carcinoma line, RPMI 2650 [7]. These variants express 200-fold increased levels of *ASS* mRNA as compared to the parental cells [8]. The canavanine-resistant variants have increased ASS activities, and are presumably resistant to canavanine because of increased conversion of citrulline to arginine. The post-transcriptional regulation identified in the canavanine-resistant variants may have physiological relevance since it has the advantage of being faster than transcriptional regulation. One would image that under particular circumstances such as inflammation, cells may employ such a mode of regulation to produce higher levels of *ASS* mRNA to meet the need for NO production. It is worth noting that similar canavanine-resistant cells have been isolated from lymphoblasts [9].

**Figure 1:**
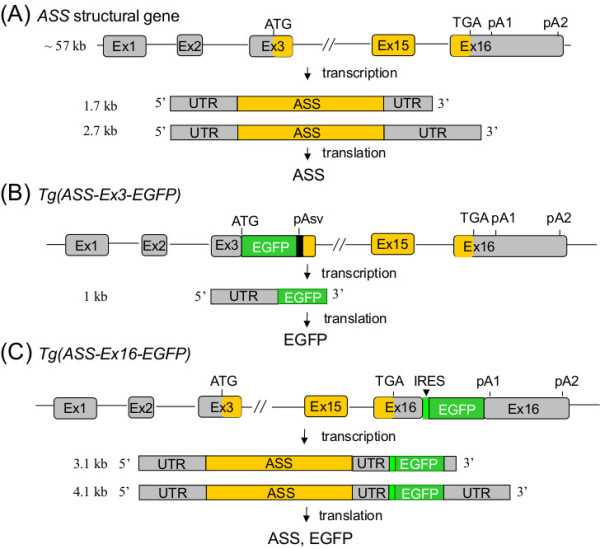
**Schematic representation of the human *ASS *gene and the *ASS-EGFP *transgene. (A)** Structure of the human *ASS* gene. Human *ASS* gene composes of 16 exons with the initiation codon (ATG) residing in exon 3 and the termination codon (TGA) in exon 16. There are two poly(A) signals, pA1 and pA2, residing in exon 16 to produce *ASS* mRNA of 1.7 kb and 2.7 kb. UTR: untranslated region. **(B)** Structure of the *Tg(ASS-Ex3-EGFP)* transgene. The *EGFP* coding sequence is inserted right after the initiation codon of the *ASS* gene. Transcription initiated from the *ASS* promoter is terminated by a SV40 poly(A) signal, pAsv, residing immediate downstream of the *EGFP* gene. EGFP but not ASS protein is produced from this transgene. **(C)** Structure of the *Tg(ASS-Ex16-EGFP)* transgene. *EGFP* is inserted in exon 16 between the termination codon and pA1 of the *ASS* gene. Depending on which poly(A) signal is used, bicistronic mRNAs of 3.1 kb and 4.1 kb are produced. A sequence of IRES (internal ribosome entrance site) is inserted in front of *EGFP* to facilitate EGFP protein translation. Both ASS and EGFP proteins are produced from this transgene.

To identify cellular targets or events that employ such regulation, a suitable *ASS* expression profiling system is essential. To this end, we took BAC (bacterial artificial chromosome) containing the entire human *ASS* structural gene of 57 kb flanked by 97 kb and 16 kb of genomic sequences at its 5′- and 3′-ends, respectively, as a starting construct to knock-in the *EGFP* (enhanced green fluorescence protein) coding sequence. Two types of *ASS-EGFP* transgene were constructed (Figure 1). One is designated as a transcription reporter, *Tg(ASS-Ex3-EGFP)*. In such a construct, *EGFP* is knocked in at a position of authentic initiation codon residing in exon 3 of the *ASS* gene where *EGFP* transcription termination is regulated by a SV40 poly(A) signal. The EGFP level in this construct would, therefore, essentially reflect the transcription activities of the *ASS* gene. Another one is designated as a transcription/post-transcription couple reporter, *Tg(ASS-Ex16-EGFP)*, in which the *EGFP* knock-in sequence also contains the sequence of the internal ribosome entry site (IRES), and the hybrid sequence is inserted into exon 16, the terminal exon of the *ASS* gene, at site between the *ASS* stop codon and the polyA signal. In such a configuration, the human *ASS* and the *EGFP* genes are transcribed as a bicistronic RNA, and *EGFP*, the downstream cistron, is translated by the IRES mechanism. Thus, EGFP activity expressed from such a transgene is subjected to both transcriptional and post-transcriptional regulation as that of the endogenous *ASS* mRNA. Using transgenic mice carrying these *ASS-EGFP* reporters, we show here that location of EGFP in the liver and kidney, the major organ of ASS production, is essentially mimic that of the endogenous *Ass* gene, suggesting sufficient regulatory element(s) is included in the *ASS-EGFP* transgene.

To obtain the temporal and spatial expression profiles of *ASS* transgene, major efforts in this study are to establish the developmental expression pattern of *ASS-EGFP* in the liver, the organ for urea production, and in the intestine and kidney where arginine biosynthesis occurs. By comparing the expression patterns between *Tg(ASS-Ex3-EGFP)* that are carrying the transcription reporter and *Tg(ASS-Ex16-EGFP)* that are carrying the transcription/post-transcription couple reporter, we aim to deduce at which level the expression control acts during development. Moreover, the *ASS* transgene is of human origin. It would be of interest to know how a human gene behaved in the mouse genetic background.

## Methods

### Animals

Mice were housed in a specific pathogen-free (SPF) area of the animal room in the Taipei Veterans General Hospital and were maintained according to protocols approved by the Animal Care and Use Committee of Taipei Veterans General Hospital. The male transgenic mice of the FVB/N strain carrying the *ASS-EGFP* transgene, i.e., *Tg(ASS-Ex3-EGFP)* or *Tg(ASS-Ex16-EGFP)*, were mated with wild-type FVB/N female where parturition occurred on day 17.5 or 18.5 after conception. Progenies carrying *EGFP* transgene were identified by visualization of fluorescence of two-week old littermates by a portable fluorescence detection system. The transgenic mouse lines *Tg(ASS-Ex3-EGFP)Tsu* and *Tg(ASS-Ex16-EGFP)Tsu* have been deposited in National Laboratory Animal Center, Taiwan, and are available for researchers on requests.

### Histological study

Histological study was performed following standard protocols [10]. In brief, mice were sacrificed by anesthetized with CO_2_ and tissue collected was fixed in 4% buffered paraformaldehyde. For frozen section, after dehydration in graded sucrose solution, tissue was embedded in OCT (Optimal Cutting Temperature) compound (Tissue Tec, Sakara, Torrance, CA). Serial sections were performed with Leica cryostat (Leica Biosystems, Wetzlar, Germany) and mounted onto slides to examine EGFP expression by a fluorescence microscope. Slides were counterstained with DAPI (4′,6-diamidino-2-phenylindole) (Roche Applied Science, Indianapolis, IN). For immunohistochemical studies, tissues fixed in buffered paraformaldehyde and embedded in paraffin were deparaffinized, hydrated in graded ethanol, and heated in 10 mM sodium citrate (pH 6.0) plus 0.1% NP-40 by microwave oven to retrieve the antigens. The remaining activity of endogenous peroxidase activity was quenched with hydrogen peroxide. After blocking, the slides were incubated with a primary antibody of GFP (1:100 dilution, anti-GFP, rabbit polyclonal antibody) (Chemicon, Billerica, MA) or 1:100 dilution of a mouse monoclonal anti-ASS antibody (BD Biosciences, San Jose, CA) overnight at 4°C. Subsequently, the slides were incubated with the biotinylated secondary antibody and streptavidin conjugated-HRP (horseradish peroxidase). The HRP was then visualized by the application of substrate chromogen DAB (diaminobenzidine) (Dako, Glostrup, Denmark) to give brown color where the slides were counterstained with hematoxylin.

### RNA isolation and S1 nuclease mapping analysis

For total RNA isolation, tissue was first grinded to powder cooled in liquid nitrogen. The frozen powder was transferred to a MagNA Lyser tube (Roche Applied Science, Indianapolis, IN) filled with 1 ml TRIzol (Invitrogen, Carlsbad, CA) and homogenized immediately by MagNA Lyser homogenizer (Roche Applied Science, Indianapolis, IN). The supernatant was used in RNA isolation following manufacturer’s instructions. For S1 nuclease mapping, appropriate restriction enzyme-digested DNA fragment was labeled at the 5’-end with [γ-^32^P] ATP and T4 polynucleotide kinase [11]. The labeled DNA probe was hybridized to total RNAs prepared from the mouse tissues. The DNA-RNA hybrids that resisted to S1 nuclease digestion were electrophoresed through a 4% polyacrylamide gel containing 7 M urea. Gel was dried and analyzed either by autoradiography or by a phosphorimager (Molecular Dynamics, Sunnyvale, CA).

## Results and discussion

### ASS-EGFP expression during liver development

**Figure 2:**
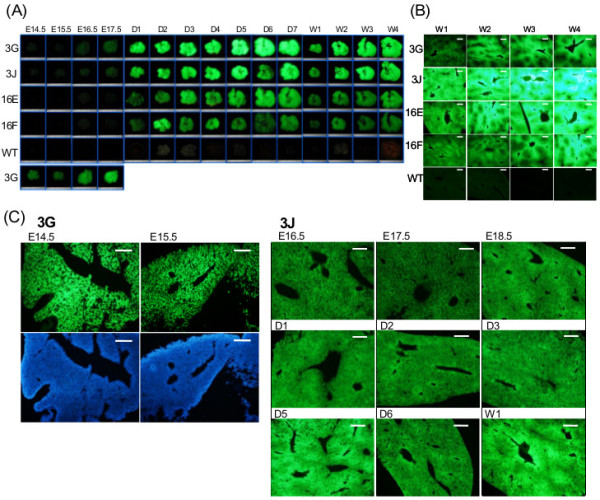
**EGFP expression during liver development in the transgenic mice. (A)** Observing EGFP fluorescence under a fluorescence dissecting microscope. Liver samples taken from fetal stages (E14.5-E17.5), 1 day to 7 day (D1-D7) and 1 to 4 week (1 W-4 W) after birth were examined directly under a fluorescence dissecting microscope. 3G, 3J, 16E, 16 F, WT represent *Tg(ASS-Ex3-EGFP) 3GTsu*, *Tg(ASS-Ex3-EGFP) 3JTsu*, *Tg(ASS-Ex16-EGFP) 16ETsu*, *Tg(ASS-Ex16-EGFP) 16FTsu* and wild-type mouse lines, respectively. Images were taken with an exposure time of 4 seconds except for 3G line with 1 second. Images in the bottom panel of fetal stage of 3G line were taken with exposure time of 4 seconds. Images were captured with magnification of 1.25-fold for samples at E14.5 to D7 and 0.7-fold for W1 to W4. Images D7 and W1 were taken from an identical sample. Each grid is 1 mm. **(B)** Comparison of liver zonation patterns among mouse lines. Liver frozen sections taken from 1- to 4-week transgenic lines and wild type were examined by fluorescence microscopy. Exposure time of the images was 50 ms except for 3G of 1 ms. Scale bar, 200 μm. **(C)** Study of EGFP fluorescence pattern in cryosections. Mouse liver sections were fixed and embedded in OCT compound. Serial frozen sections were examined by fluorescence microscopy. Left bottom panel was sections stained by DAPI. The liver stage and mouse line employed are as indicated. Scale bar, 200 μm.

ASS expression in the liver has been shown to be developmentally regulated. In the rat, *Ass* mRNA is first detected in E15.5 fetal stage and increases continuously to neonatal stage [12]. To visualize ASS expression during liver development and to study whether post-transcriptional regulation is involved, studies were taken using transgenic mice *Tg(ASS-Ex3-EGFP) 3GTsu* and *Tg(ASS-Ex3-EGFP) 3JTsu*, abbreviated as 3G and 3J, that carry, respectively, 30 and 2 copies of the transcription reporter and *Tg(ASS-Ex16-EGFP) 16ETsu* and *Tg(ASS-Ex16-EGFP) 16FTsu*, abbreviated as 16E and 16 F, that carry, respectively, 10 and 5 copies of the transcription/post-transcription couple reporter. EGFP fluorescence of liver tissues taken from fetuses at E14.5 to E17.5 (some to E18.5), 1 to 7 days and 2, 3 and 4 weeks after birth were examined directly under a fluorescence dissecting microscope (Figure 2A). Images were captured with 1.25-fold magnification for samples at stages E14.5 to D7 and 0.7-fold for those at stages W1 to W4. Images D7 and W1 in Figure 2A were taken from an identical sample. The difference in their fluorescence brightness is mainly due to the fact that the image brightness is inversely proportional to the square of the transverse magnification [13]. Moreover, image was captured using a constant 4-second exposure time except in 3G line which was reduced to 1 second due to strong EGFP signals. At E14.5 stage, EGFP fluorescence was weak except in the 3G line (Figure 2A, bottom panel, exposed for 4 seconds). Considerable enhancement of EGFP signal could be seen at day 1 and increased gradually to 4 weeks. Similar patterns of expression could also be seen in the 16E and 16 F lines, except that the signals were weaker (Figure 2A). Variations in EGFP fluorescence among mouse littermates were also observed. For example, the EGFP fluorescence was relatively strong in a liver sample of 16 F (Figure 2A, 16 F D2).

Zonation of metabolic pathways is believed to be a mechanism leading to efficient use of precursor pools and energy in an organism [14]. In the rodent, urea cycle enzymes in the liver show zonation distribution, i.e., they are present predominantly in the periportal hepatocytes and declining gradually toward the pericentral hepatocytes. Such a distribution pattern occurs not only at the protein level but also in the mRNAs of these enzymes [14]. To study whether zonation can be visualized by EGFP fluorescence, frozen sections of the liver were examined. Similar EGFP zonation pattern could clearly be seen between transgenic lines carrying the transcription reporter, i.e., 3G and 3J, and the transcription/post-transcription couple reporter, i.e., 16E and 16 F (Figure 2B). In the rodent, *Ass* RNA zonation has been shown to appear 2 days before birth [14]. In agreement, zonation gradually became apparent at E17.5 and E18.5 (Figure 2C, 3J line) where near-homogeneous EGFP fluorescence distribution was found at E14.5 and E15.5 stages (Figure 2C, 3G line). However, human ASS has been reported to be present in all hepatocytes with no marked zonation [15]. The *ASS-EGFP* transgene in this study is of human origin, and yet EGFP expression manifested zonation pattern similar to that of the rodent *Ass*. Apparently, establishment of zonation pattern is made at transcription initiation and such regulation may be influenced by cellular environment. Thus, human *ASS* gene when in the rodent background follows the expression pattern of the rodent.

### ASS-EGFP expression during kidney development

**Figure 3:**
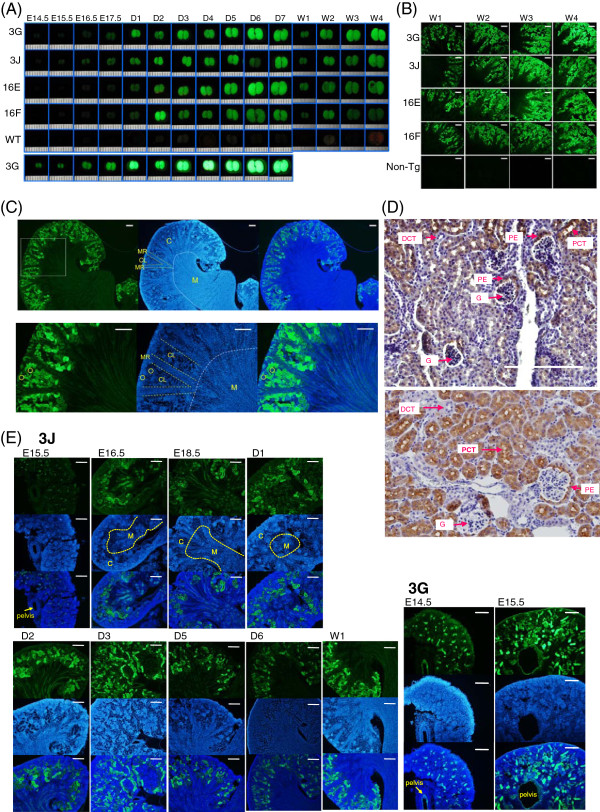
**EGFP expression during kidney development in the transgenic mice. (A)** Study of EGFP fluorescence by fluorescence dissecting microscopy. Conditions were as described in the legends of Figure 2A. **(B)** Comparison of fluorescence patterns of the kidney among the mouse lines. Kidney frozen sections taken from 1- to 4-week transgenic mice and non-Tg controls were examined by fluorescence microscopy. Exposure time was 50 ms except for 3G of 1 ms. Scale bar, 200 μm. **(C)** Localization of EGFP fluorescence. Frozen sections of kidneys from 1-week old transgenic mouse 3J were examined by fluorescence microscopy. A region of enlarged image was selected (white box) and shown in the lower panel. In each panel: left, fluorescence image; middle, DAPI staining; right, fluorescence image merged with DAPI staining. Particular regions are distinguished by dotted lines: C: cortex; M: medulla; CL: cortical labyrinth; MR: medullary ray. The glomerulus is circled in yellow. **(D)** Immunohistochemical localization of EGFP and ASS. Paraffin sections of the kidney from 4 week-old transgenic mouse 3G were incubated with an EGFP antibody (upper panel) or ASS antibody (lower panel). The slides were incubated with the biotinylated secondary antibody coupled with streptavidin conjugated-HRP and was visualized by the application of chromogen DAB to produce brown color when the slides were counterstained with hematoxylin. PCT: proximal convoluted tubule; DCT: distal convoluted tubule; G: glomerulus; PE: parietal epithelium. Scale bar, 200 μm. **(E)** EGFP fluorescence patterns during kidney development of the transgenic mice. Mouse kidney was fixed and embedded in OCT compound. Serial frozen sections were examined by fluorescence microscopy. In each panel: top, EGFP fluorescence image, middle, DAPI staining, bottom, EGFP fluorescence image merged with DAPI staining. The developmental stages and mouse lines employed are as indicated. Areas of the cortex (C) and the medulla (M) are distinguished by dotted lines. Scale bar, 200 μm.

Kidney is the major organ of arginine production in adult where ASS is the rate-limiting step in the conversion of citrulline to arginine [1]. Similar to the liver, EGFP fluorescence in the kidney was weak at E14.5 stage except that of the 3G line (Figure 3A, bottom panel). Marked enhancement of EGFP expression was observed during neonatal (Figure 3A, D1 stage). The ASS protein and mRNA have been shown to localize exclusively in the proximal tubules of renal cortex [16]. Indeed, in our transgenic mice, EGFP fluorescence was mainly found in the renal cortex area (Figure 3B). Renal cortex can be divided into alternate regions of cortical labyrinth and the medullary rays [17]. EGFP signal appeared in cortical labyrinth (Figure 3C, CL) and not in the medullary rays (Figure 3C, MR). The cortical labyrinth consists of glomeruli and convoluted tubules where EGFP signal appeared in the convoluted tubules and not in the glomeruli (Figure 3C, lower panel). To examine the kidney structure in detail, immunohistochemical studies using EGFP and ASS antibodies were performed (Figure 3D). Signals were found in the proximal convoluted tubules and the parietal epithelium but were absent in the glomeruli and the distal convoluted tubules when analyzed by either EGFP or ASS antibodies. Therefore, the *ASS-EGFP* transgene is faithfully expressed as an authentic *ASS* gene. On the other hand, to study the EGFP expression pattern during kidney development, kidney frozen sections in various developmental stages were examined (Figure 3E). At E14.5 and E15.5 stages, EGFP fluorescence was found to distribute rather homogenously in the entire kidney (Figure 3E, 3G line). Fluorescence in the structure of the renal pelvis was also visible. The nephrogenic zone can be divided into the cortex and medulla in mouse kidney at E15.5 [18]. During perinatal stage, EGFP fluorescence was found primarily in the cortex although weak signals were visible in the collecting duct of the medulla (Figure 3E, stages E16.5-D2). At later stages, fluorescence was essentially seen in the cortex where areas of fluorescence increased. Thus, *ASS-EGFP* expression recaptures the feature of *Ass* gene in the mouse kidney [16].

### ASS-EGFP expression during intestine development

**Figure 4:**
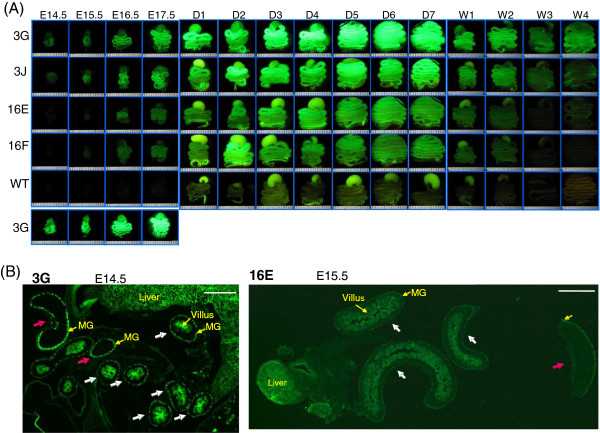
**EGFP expression during intestine development in the transgenic mice. (A)** Study of EGFP fluorescence by fluorescence dissecting microscopy. At stage E14.5 to 2 week, structures including the stomach, small and large intestines were included and examined. Due to the size, at the 3-week stage only stomach and small intestine and at 4 weeks small intestine alone were examined. Conditions were as described in the legends to Figure 2A. **(B)** Expression of EGFP during small intestine development. Fetuses at E14.5 stage of 3G line (left panel) and E15.5 stage of 16E line (right panel) were embedded in OCT compound. Frozen sections were examined by fluorescence microscopy. White arrow marked the proximal intestine and red arrow marked the distal intestine. MG: myenteric ganglion. Scale bar, 500 μm.

The small intestine is the major organ in arginine synthesis during the perinatal stage of development, but gradually shifts to citrulline production. Citrulline generated is then taken up by the kidney where it is converted to arginine. The collaboration between the intestine and the kidney in arginine synthesis becomes established postnatally, constituting the intestinal-renal axis [19]. To study whether EGFP fluorescence in the small intestine in the *ASS-EGFP* transgenic mice can reproduce such regulation, EGFP fluorescence in the digestion system was examined directly under a fluorescence dissecting microscope (Figure 4A). In contrast to the liver and kidney, the EGFP signals in the intestine were clearly visible in the fetal stages (Figure 4A). The signals persisted in to the perinatal stage but decreased gradually around the time of weaning (Figure 4A). It is noted that substantial auto-fluorescence could be seen in organs of a wild-type mouse, especially in the stomach (Figure 4A, WT). Such signals in part originated from the food intake (data not shown). On the other hand, De Jonge et al. [20] have studied temporal expression of urea cycle enzymes in the rat small intestine during perinatal development. By northern blot analysis and in situ hybridization, they found in the fetal stage that the proximal loops of the intestine expressed the *Ass* mRNAs at higher concentrations than the more distal loops. In agreement, frozen sections of the transgenic fetuses at E14.5 or E15.5 showed pronounced EGFP signals in the proximal loops of the intestine in the region closer to the liver (Figure 4B, white arrow) while only weak signals were seen in the distal parts of the intestine (Figure 4B, red arrow). In addition, EGFP signals were clearly seen in the enteric neurons of the myenteric ganglion (Figure 4B, MG). Thus, EGFP signals in the small intestine of the *ASS-EGFP* transgenic mice appear to recapture the temporospatial expression of rodent *Ass*.

### Expression profiling of EGFP and Ass mRNAs in the liver, kidney and intestine by S1 nuclease mapping

**Figure 5:**
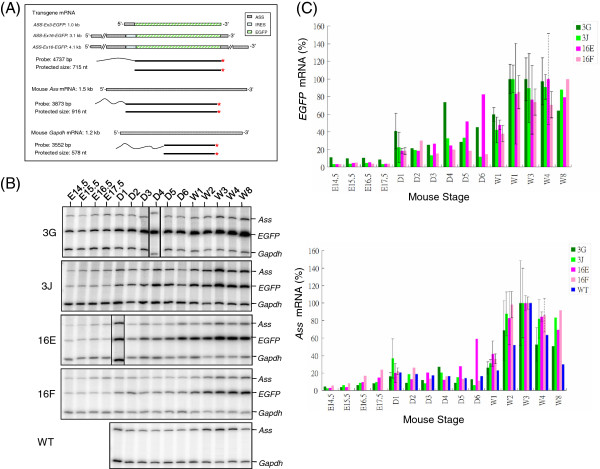
**Determination of relative abundances of *EGFP *and mouse *Ass *mRNAs during liver development by S1 nuclease mapping. (A)** Strategy of S1 nuclease mapping. RNA structures, DNA probes and the predicted protected probe size after S1 nuclease digestion are depicted. Stars indicate ^32^P 5*'*-end labels; wavy line at one end of the labeled probe indicates vector sequences that distinguish a re-annealed probe from a probe that was protected by hybridization to RNA. **(B)** Phosphorimage analysis of the protected fragments from S1 nuclease mapping. RNAs isolated from various stages of liver development (indicated on top) were hybridized to the 5*'* end-labeled probes and the DNA-RNA hybrids that resisted S1 nuclease digestion were electrophoresed through a 4% polyacrylamide gel containing 7 M urea. Gel was dried and analyzed by a phosphorimager. The protected fragments corresponding to mRNAs of mouse *Ass*, *EGFP* transgene and mouse *Gapdh* are marked on the right. Mouse lines from which RNAs were obtained are indicated on the left. **(C)***EGFP* and *Ass* mRNA profiles during liver development. The intensities of hybridization signals of *EGFP*, *Ass* and *Gapdh* mRNAs were quantified by a phosphorimager where the levels of *EGFP* and *Ass* mRNAs were normalized to the level of the *Gapdh* mRNA. Relative abundances of *EGFP* mRNA (upper panel) and *Ass* mRNA (lower panel) during liver development are plotted by taking the stage of the highest level as 100%. Standard deviation (solid line) is shown if analysis was performed at least 3 times. If performed twice, average with interval of two values is shown (dotted line).

**Figure 6:**
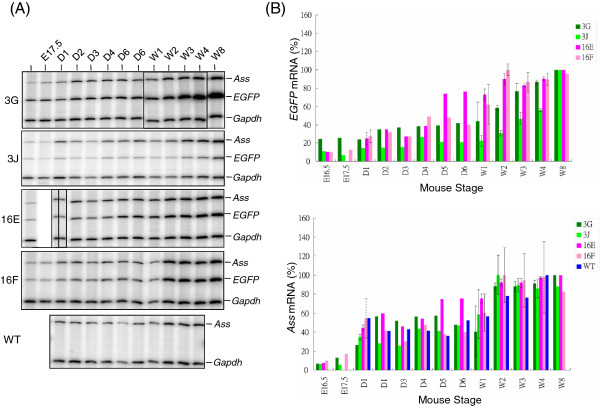
**Determination of relative abundances of *EGFP *and mouse *Ass *mRNAs during kidney development by S1 nuclease mapping. (A)** Phosphorimage analysis of the protected fragments from S1 nuclease mapping of kidney RNA. Condition was as described in the legends to Figure 5. **(B)***EGFP* and *Ass* mRNA profiles during kidney development. The intensities of hybridization signals of *EGFP*, *Ass* and *Gapdh* mRNAs were quantified by a phosphorimager where the levels of *EGFP* and *Ass* mRNAs were normalized to the level of the *Gapdh* mRNA. Relative abundances of *EGFP* mRNA (upper panel) and *Ass* mRNA (lower panel) during kidney development are plotted by taking the stage of the highest level as 100%. Standard deviation (solid line) is shown if analysis was performed at least 3 times. If performed twice, average with interval of two values is shown (dotted line).

**Figure 7:**
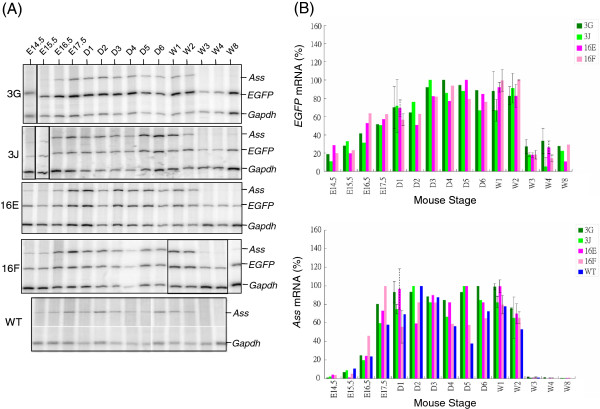
**Determination of relative abundances of *EGFP *and mouse *Ass *mRNAs during intestine development by S1 nuclease mapping. (A)** Phosphorimage analysis of the protected fragments from S1 nuclease mapping of intestine RNA. Condition was as described in the legends to Figure 5. **(B)***EGFP* and *Ass* mRNA profiles during intestine development. The intensities of hybridization signals of *EGFP*, *Ass* and *Gapdh* mRNAs were quantified by a phosphorimager where the levels of *EGFP* and *Ass* mRNAs were normalized to the level of the *Gapdh* mRNA. Relative abundances of *EGFP* mRNA (upper panel) and *Ass* mRNA (lower panel) during kidney development are plotted by taking the stage of the highest level as 100%. Standard deviation (solid line) is shown if analysis was performed at least 3 times. If performed twice, average with interval of two values is shown (dotted line).

Studies described above show that EGFP fluorescence in the liver, kidney and intestine examined by fluorescence microscopy either directly or after cryosectioning is useful to study *ASS* expression. In such analyses, no gross disparity in EGFP expression was found between mouse lines of *Tg(ASS-Ex3-EGFP) Tsu* and *Tg(ASS-Ex16-EGFP) Tsu*. To be quantitative, *EGFP* mRNA profiles of the liver, kidney and intestine were examined by S1 nuclease mapping. In addition, the *ASS-EGFP* transgene is of human origin. To examine whether species-specific expression pattern existed, comparison was also made between expression profiles of the *EGFP* mRNA and the endogenous mouse *Ass* mRNA. Abundances of *EGFP* and *Ass* mRNAs from the liver, kidney and intestine in different developmental stages were analyzed and quantified by S1 nuclease mapping; where levels of *EGFP* and *Ass* mRNAs were first normalized to the mRNA level of *Gapdh*, a house keeping gene glyceraldehyde-3-phosphate dehydrogenase. The mRNA structures of *EGFP*, *Ass* and *Gapdh,* their 5′-end labeled cDNA probes and the expected sizes of protected fragment are depicted in Figure 5A. The probes are designed such that the size of protected fragment by respective probes could easily be distinguished by denatured polyacrylamide gel electrophoresis. RNAs were hybridized in reaction containing individually end-labeled cDNA probes of *EGFP*, mouse *Ass* and mouse *Gapdh*. DNA-RNA duplexes were analyzed by electrophoresis after removing unhybridized RNA and single-stranded probes by S1 nuclease. It is worth noting that in this analysis, the human *ASS* mRNA expressed from the *Tg(ASS-Ex16-EGFP) Tsu* line cannot be detected by the mouse *Ass* probe. Moreover, to study RNA expression of a particular organ, same batch of the labeled probes was used to avoid signal variations due to differences in probe specific activities. Representative images acquired by the phosphorimager on the analysis of liver, kidney and intestinal RNAs and the expression profiles of *EGFP* and *Ass* mRNAs are presented (Figures 5, 6 and 7).

Analysis of liver RNA from fetal E14.5 stage to 4 week after birth, *EGFP* mRNA level was found to increase progressively during the development (Figure 5B). Significant variations in *EGFP* mRNA abundances among mouse littermates were found. Such variations were also noted when EGFP fluorescence of a particular organ was examined directly (Figures 2A, 3, 4A). To study whether EGFP expression profile differs between *Tg(ASS-Ex3-EGFP) Tsu* and *Tg(ASS-Ex16-EGFP) Tsu* lines during liver development, relative abundances of *EGFP* mRNAs was determined (Figure 5C, upper panel). The *EGFP* mRNA levels among the transgenic lines were found to be approximately reflecting their transgene copy numbers (Figure 5C, upper panel), suggesting that developmental *ASS* expression in the liver is mainly controlled at transcription initiation. If the novel post-transcriptional regulation affecting *ASS* nuclear precursor RNA stability similar to that in canavanine-resistant variants occurred in the *Tg(ASS-Ex16-EGFP) Tsu* line [7], one would expect the *EGFP* mRNA level in the *Tg(ASS-Ex16-EGFP) Tsu* line to increase by one to two orders of magnitude from current levels. However, whether any subtle post-transcriptional regulation on *ASS* mRNA has taken place would require further studies. Nevertheless, both the 1.7- and 2.7-kb *ASS* mRNA species are highly stable with a half-life of approximately 15–20 h [21]. It is noted that among the organs examined, EGFP fluorescence in the *Tg(ASS-Ex16-EGFP) Tsu* line was generally weaker yet considerably high levels of *EGFP* mRNA could be detected. It is likely that *EGFP* in the *Tg(ASS-Ex16-EGFP) Tsu* line, which is the downstream cistron, is translated by IRES mechanism which may be less efficient comparing to cap-dependent translation of *EGFP* in the *Tg(ASS-Ex3-EGFP) Tsu* line.

In rodents, *Ass* gene expression, assayed by RNA or by activities, is reported to increase progressively towards birth reaching about 50% of adult liver value [1]. In contrast, in human fetuses, ASS activities reached 90% of the adult value at 36 weeks of gestation [22]. Our study showed that the *EGFP* mRNA abundances at birth were less than 40% of the adult values (Figure 5C, upper panel). Thus, *EGFP* mRNA profile during liver development is similar to that of mouse *Ass* mRNA, suggesting that the human *ASS* gene in the mouse background follows the mouse *Ass* developmental pattern. Similar conclusion has been obtained as evidenced by liver zonation distribution of EGFP fluorescence (Figure 2B). Thus, the human *ASS* gene in the mouse genetic background follows mouse *Ass* developmental pattern. This is not surprising since the *ASS* gene during liver development is known to subject to both hormonal and nutritional regulation [1,2]. Therefore, cellular environment plays important role in *ASS* expression. The results indicate that the *cis*-elements required for *ASS* expression during liver development are similar between the human and the mouse, and the *ASS-EGFP* transgene carries sufficient elements to execute such regulation.

To study whether the transgene expression may affect endogenous *Ass* gene expression, levels of *Ass* mRNA were compared between the 3G and 3J lines of *Tg(ASS-Ex3-EGFP) Tsu*, which carry 30 and 2 copies of transgene, respectively. Indeed, a significantly higher level of *EGFP* mRNA was found in the 3G line compared to that of the 3J line, yet comparable abundance of *Ass* mRNA was detected among the transgenic lines 3G and 3J and that of the wild-type mouse (Figure 5C, lower panel). Similar results were found in the analysis of the *Tg(ASS-Ex16-EGFP) Tsu* line (Figure 5C, lower panel 16E and 16 F). Therefore, even in the presence of high copies of the transgene, no apparent sequestration of transcription factors to affect endogenous gene expression occurs.

Approach similar to that of the liver was taken to study *EGFP* mRNA expression during kidney development (Figure 6). However, due to limit amounts of sample in fetal E14.5 and E15.5 stages, analysis started at fetus E16.5 stage. Representative images of RNA analysis by S1 nuclease mapping are shown in Figure 6A. The EGFP expression profile was similar between the *Tg(ASS-Ex3-EGFP) Tsu* and *Tg(ASS-Ex16-EGFP) Tsu* lines (Figure 6B, upper panel), suggesting *ASS* expression during kidney development is controlled mainly at the transcription level. By comparing *Ass* mRNA levels between wild-type and transgenic lines, one concludes that expression of the transgene does not interfere with expression of the endogenous *Ass* gene during kidney development in the mouse (Figure 6B, lower panel).

To study *EGFP* mRNA expression profiles in the intestine, RNA of the digestion system including stomach to rectum at the fetal stage was isolated. After birth, only RNA from the small intestine was analyzed. Because EGFP fluorescence intensity was found to vary among different sections of the small intestine (data not shown), to avoid complication, the entire small intestine was collected and used for RNA isolation. In this study, we again showed that *EGFP* mRNA abundance during intestine development is mainly controlled at the transcription initiation and expression of the transgene did not interfere with endogenous *Ass* expression (Figure 7). Interestingly, although there were similarities between the expression profiles of *EGFP* and *Ass* mRNAs, some differences did exist (Figure 7B). For example, in contrast to *EGFP* mRNA which showed smooth increases in its abundance perinatally, a rather sharp increase in *Ass* mRNA abundance at E17.5 stage was observed (Figure 7B). Moreover, the *Ass* mRNA levels declined sharply at 3 weeks of age, but substantial *EGFP* mRNA could be detected at this stage in both *Tg(ASS-Ex3-EGFP) Tsu* or *Tg(ASS-Ex16-EGFP) Tsu* lines (Figure 7B). In this regard, a previous study of arginine-metabolizing enzymes in the developing rat small intestine found that mRNA levels of all genes in arginine metabolism were highest during the suckling period where *Ass* mRNAs declined to hardly detectable levels in the second postnatal week [20]. On the other hand, in piglets, net synthesis of arginine declines more gradually in the small intestine and is still present at 7 weeks of age [23]. The perinatal human intestine resembles that of piglets in that ASS activities are highest during the suckling period and declines to low levels around weaning and then rises again [24]. Thus, in contrast to mouse *Ass* mRNA that disappears completely at 3 week, the *EGFP* mRNA expression profile of the transgenic mice resembles human *ASS* in that substantial levels of *EGFP* mRNA could still be detected at 8 weeks of age (Figure 7). Apparently, *ASS* gene in humans and pigs has specific *cis*-element(s) which differ from those of the rodent in regulating *ASS* expression in the small intestine. By comparison of the upstream *ASS* gene sequences in human, pig and mouse, such *cis*-element(s) may be deduced.

Using transgenic mouse system, we show in this study that developmental- and tissue-specific *ASS* expression of liver, kidney and intestine is mainly controlled at transcription initiation. This is not surprising since transcription initiation, the first step of gene regulation, is the most important mechanism to determine whether or not genes are expressed and then, how much of encoded mRNAs are produced. On the other hand, the novel post-transcriptional regulation identified in canavanine-resistant variants may be so genetically programmed in particular cell types to meet immediately the demand for high ASS activity. Our preliminary study indicates such regulation may occur during mouse fetal brain development (T Su, unpublished data). The region of the brain involved is currently under characterization. On the other hand, the *ASS* gene in this transgenic mouse system is of human origin. How would the transcriptional programs of human gene follow in mouse genetic background? In this regard, Wilson et al., [25] have initiated an important study by taken a mouse model of Down syndrome in which mouse cells contain a copy of human chromosome 21. They concluded that genetic sequence is largely responsible for directing transcriptional programs in homologous tissues. Others such as interspecies differences in epigenetic machinery, cellular environment, and transcription factors themselves play secondary roles. Our study shows that developmental program of *ASS-EGFP* transgene in liver is similar to rodent, suggesting genetic elements determining liver development are comparable between human and rodent. Thus, cellular environments play important role in shaping *ASS-EGFP* transcriptional program during liver development. On the other hand, the time course of *EGFP* expression in small intestine resembles that of human *ASS* gene, suggesting the presence of particular genetic sequence(s) in *ASS* gene that dictates human intestine development.

## Conclusions

We demonstrate that this transgenic mouse system is ideal for annotation of temporal and spatial expression profiles of the *ASS* gene. In particular, the *Tg(ASS-Ex3-EGFP) 3GTsu* line, containing 30 copies of the transgene, generates strong EGFP signals, and is, thus, useful in revealing weak expression. It is conceivable that a comprehensive knowledge of cell types expressing ASS may provide insights into the function. Such knowledge should facilitate investigation of the role of ASS in conditions of physiological and disease states, especially when *ASS* mRNA or protein are found not to be expressed in many tumours including hepatocellular carcinoma (HCC), melanoma, some mesotheliomas, renal cell cancers, sarcomas and lymphomas [26]. As a result, arginine deprivation employing the pegylated form of arginine deiminase (ADI-PEG20) as a targeted therapy is currently in clinical trials for patients with HCC and melanoma [26]. In this respect, feature of down-regulated expression of ASS in HCC has been recaptured in our *ASS-EGFP* transgenic mouse model (manuscript in preparation). Thus, questions such as physiological or pathophysiological response of ASS expression stimulated by a variety of signals may be tackled using this system.

## Competing interests

The authors declare that they have no competing interests.

## Authors’ contributions

SS carried out the experiments. MH participated in mouse study. TS conceived of the study, participated in its design and writing the manuscript. All authors read and approved the final manuscript.
